# Bedrests—A Sad Though Simple Story

**Published:** 1888-06-09

**Authors:** 


### BEDRESTS—A SAD THOUGH SIMPLE STORY.

Might I, through the medium of your paper, beg some kind friends to give me a helping hand towards getting a couple of "bedrests" for my poor patients? The only rest I can boast of is a chair, which I cover with pillows, not always too comfortable; and not having too many chairs or pillows, cannot manage always to give "rests" to as many as really need them. We have a sad case at present in hospital, a woman suffering from paralysis of the left side. We admitted her in March. When she had been with us about four weeks, her husband (the only bread-winner of the family) got cold, and continued his work till forced by rheumatism to give in. He then applied to our doctor and was sent in to us. Rheumatic fever followed, and he is still unable to even dress or feed himself, let alone work for his family. The children are now applying to the parish for relief. Our hospital is not a free one; the patients are supposed to pay 5*s.* and upwards, but such cases as I have just mentioned cannot be turned away from our doors, therefore our voluntary contributions get used up taking in patients, leaving little or none towards getting such articles as I am now begging for. W. S.

Axminster Hospital, Devon.

